# Whole-brain modeling of the differential influences of amyloid-beta and tau in Alzheimer’s disease

**DOI:** 10.1186/s13195-023-01349-9

**Published:** 2023-12-05

**Authors:** Gustavo Patow, Leon Stefanovski, Petra Ritter, Gustavo Deco, Xenia Kobeleva, for the Alzheimer’s Disease Neuroimaging Initiative

**Affiliations:** 1 ViRVIG, Universitat de Girona, Girona, Spain; 2 Department of Information and Communication Technologies, Universitat Pompeu Fabra, Center for Brain and Cognition, Computational Neuroscience Group, Barcelona, Spain; 3 Berlin Institute of Health at Charité – Universitätsmedizin Berlin, Berlin, Germany; 4 Department of Neurology with Experimental Neurology, Brain Simulation Section, Charité – Universitätsmedizin Berlin, corporate member of Freie Universität Berlin and Humboldt-Universität zu Berlin, Berlin, 10117 Germany; 5 Bernstein Center for Computational Neuroscience, Berlin, Germany; 6 Einstein Center for Neuroscience Berlin, Berlin, Germany; 7 Einstein Center Digital Future Berlin, Berlin, Germany; 8 Institució Catalana de la Recerca i Estudis Avançats (ICREA), Barcelona, Spain; 9 Computational Neurology Research Group, Ruhr University Bochum, Bochum, Germany; 10 German Center for Neurodegenerative Diseases (DZNE), Bonn, Germany; 11 Clinic for Neurology, University Hospital Bonn, Bonn, Germany

**Keywords:** Alzheimer’s disease, Amyloid-beta, Tau, Whole-brain model, Simulation

## Abstract

**Background:**

Alzheimer’s disease is a neurodegenerative condition associated with the accumulation of two misfolded proteins, amyloid-beta (A$\beta$) and tau. We study their effect on neuronal activity, with the aim of assessing their individual and combined impact.

**Methods:**

We use a whole-brain dynamic model to find the optimal parameters that best describe the effects of A$\beta$ and tau on the excitation-inhibition balance of the local nodes.

**Results:**

We found a clear dominance of A$\beta$ over tau in the early disease stages (MCI), while tau dominates over A$\beta$ in the latest stages (AD). We identify crucial roles for A$\beta$ and tau in complex neuronal dynamics and demonstrate the viability of using regional distributions to define models of large-scale brain function in AD.

**Conclusions:**

Our study provides further insight into the dynamics and complex interplay between these two proteins, opening the path for further investigations on biomarkers and candidate therapeutic targets in-silico.

**Supplementary Information:**

The online version contains supplementary material available at 10.1186/s13195-023-01349-9.

## Background

Alzheimer’s disease (AD) is a neurodegenerative disease that leads to progressive impairment of memory and other cognitive domains, neuropsychiatric symptoms, and, ultimately, severe impairment in all body functions. This results in both a large loss of quality of life for affected people and caregivers and high costs for society at large. AD pathogenesis is associated with several interlinked pathomechanistic processes, from genomics to connectomics, including the Notch-1 pathway, neurotransmitters, polygenetic factors, neuroinflammation, and neuroplasticity [1]. However, the accumulation of misfolded proteins is considered to be the pathological hallmark of AD: namely extracellular accumulation of Amyloid-beta (A$\beta$), forming senile plaques; and intraneuronal aggregation of the microtubule protein tau, called neurofibrillary tangles [2]. Treatments for removal of A$\beta$ (e.g., with Adacanumab and Lecanemab) are currently discussed in light of inconclusive effects on reducing cognitive decline [3]. In spite of the large body of research on AD, many aspects regarding pathophysiology and the roles of A$\beta$ and tau are still incompletely understood [4, 5].

Regarding brain dysfunction, several human autopsy and animal studies have seen a disruption in excitation/inhibition (E/I) balance, especially in early stages where neuronal hyperexcitability impairs cortical activity and thus contributes to cognitive decline [6, 7]. Chang et al. [8] showed that tau affects excitatory and inhibitory neurons differently, and its removal decreases the baseline activity of excitatory neurons and, simultaneously, affects the axon initial segments and the intrinsic excitability of inhibitory neurons, resulting in network inhibition. In this line, Bi and co-workers [9] hypothesized that A$\beta$ impairs GABAergic function and thus produces synaptic hyperexcitation. Petrache et al. [10] found synaptic hyperexcitation and severely disrupted E/I inputs onto principal cells and a reduction of the somatic inhibitory axon terminals. Recently, Lauterborn and coauthors [11] found significantly elevated E/I ratios in post-mortem cortex samples. While interesting results regarding E/I imbalance with marked hyperexcitability were derived in animals and post-mortem human cortex samples, in-vivo human studies are lacking, as the activity of E/I populations cannot be directly measured using neuroimaging. Most works on whole-brain dynamics studied activation patterns but were not informative regarding the role of E/I populations [12–16]. To understand the complex interplay between pathophysiological processes and brain activity (i.e., fMRI), models might contribute more biologically plausible insights when incorporating heterogeneity of brain dynamics based on empirical data [17–19].

Earlier work using whole-brain simulations focused on linking global and local brain dynamics to individual differences in cognitive performance scores in healthy subjects and in patients with AD [12]. Demirtaş [14] et al. studied the effect of heterogeneity of local synaptic strengths on a dynamical model of the human cortex in healthy subjects, showing that heterogeneity significantly improved the fitting of resting-state functional connectivity. Stefanovski and co-authors [15] focused on the connection of A$\beta$ with neural function in The Virtual Brain [20] to examine how A$\beta$ modulates regional E/I balance, producing local hyperexcitation in regions with high A$\beta$ loads. This led to further improvements in classifications between AD and controls [16]. However, all these works studied the effect of a single burden, namely A$\beta$, on the neuronal dynamics, while our work focuses mostly on the *interplay* of both burdens, i.e., A$\beta$ and tau, assessing their relative impacts on brain dynamics.

In this paper, we use whole-brain modeling techniques to study the impact of both A$\beta$ and tau on the dynamics of regional behaviors in AD, discerning the impact of each protein in isolation and in combination, and being able to assess their relative weights on contributing to abnormal brain activity. We use the balanced excitation-inhibition (BEI) model [18], which can reproduce the fMRI activity based on interactions of excitatory and inhibitory neural populations interconnected by white matter tracts. We show in this work a clear dominance of the effects of A$\beta$ over tau on brain dynamics in the earlier stages of the disease (mild cognitive impairment, MCI), and a dominance of protein tau over A$\beta$ in advanced stages (manifest dementia).

## Methods

### Methods overview

#### Model creation

**Fig. 1:**
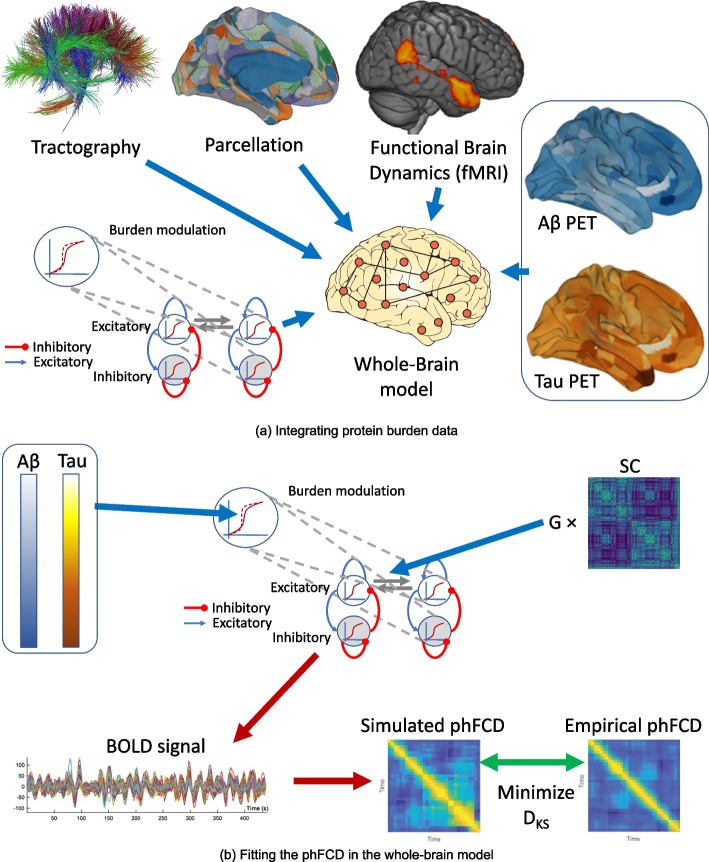
Illustrative overview of our processing pipeline. **A** Basic ingredients for the integration of protein burden data from structural (dMRI, top left), functional (fMRI, top right), and burden (PET, right) using the same parcellation for each neuroimaging modality (top, middle) for generating a whole-brain computational model (bottom left). Each node of the model is using a realistic underlying biophysical neuronal model including AMPA (blue connections), GABA (red), and NMDA (gray) synapses as well as neurotransmitter gain modulation of these. **B** Fitting the measures in the whole-brain model: First, we simulate the BOLD timeseries for each brain region in the parcellation, for each subject. These timeseries are defined by its inputs, namely a previously found global coupling constant *G*, an individual Structural Connectivity (*SC*) matrix, and the corresponding individual A$\beta$ and tau burdens. Subsequently, we compute a time-versus-time matrix of phase functional connectivity dynamics (phFCD). This is compared to a reference empirical phFCD extracted from the fMRI data off the same subject using the Kolmogorov-Smirnov distance (KS), $D_{KS}$, which is minimized to find the generative parameters of the model. This process is repeated for the other two measures of brain dynamics, functional connectivity (FC) and sliding-window functional connectivity dynamics (swFCD)

Figure 1 presents an overview of our overall analysis strategy, and the details can be found in the “Methods” section. We make use of MRI and positron emission tomography (PET) from the Alzheimer’s Disease Neuroimaging Initiative (ADNI). In summary, we use diffusion MRI to generate the structural connectomes of healthy controls (HC), mild cognitive impairment (MCI), and Alzheimer’s disease (AD) subjects. We use task-free resting-state functional MRI to fit a whole-brain model in which the local neuronal dynamics of each brain region evolve according to the dynamic mean field model by Deco et al. [18], which is then connected to a spontaneous blood-oxygenation-level-dependent (BOLD) dynamics. We refer to this model as the *balanced excitation-inhibition (BEI)* model, which can be thought of as a homogeneous reference against which we evaluate the performance of our heterogeneous AD model. A$\beta$ and tau distributions are derived from AV-45 and AV-1451 PET from ADNI. For the heterogeneous model, we incorporate regional heterogeneous distributions of the main proteins involved in AD, namely A$\beta$ and tau, as first-order multiplicative polynomials for each burden and for each type of population (excitatory/inhibitory) into the local gain parameter $M_{(E,I)}$. Fitting the model to empirical fMRI data allows us to evaluate which effect of A$\beta$ and tau on the different populations can mechanistically explain the observed behaviors.

#### Model fitting

For both of our models, homogeneous and heterogeneous, we assume that all diffusion MRI-reconstructed streamline fibers have the same conductivity, and thus the coupling between different brain areas is scaled by a single global parameter, *G*. We first tune the *G* parameter of the BEI model to adjust the strength of effective coupling in the model and identify the brain’s dynamic working point by fitting the model to three empirical properties that are estimated from the empirical fMRI data:

- The Pearson correlation between model and empirical estimates of static (i.e., time-averaged) functional connectivity estimated across all pairs of brain regions (FC);
- Similarity in sliding-window functional connectivity dynamics (swFCD); and
- The KS distance between a set of dynamic functional connectivity matrices (also called coherence connectivity matrix [21]) built from the average BOLD time series of each ROI, which were Hilbert-transformed to yield the phase evolution of the regional signals (phFCD).

We then fit the coefficients for the two burdens, for excitatory and inhibitory populations, with a global optimization algorithm, within directional bounds obtained from previous clinical observations (see below, in the “Constraints” section).

#### Result evaluation

To demonstrate that E/I imbalance is dependent on the precise distribution of the A$\beta$ and tau burdens, at the optimal values obtained with the fitting procedure described above, we randomly shuffled the empirical protein burdens; i.e., the original 378 values for each of the misfolded protein maps were randomly re-assigned to different regions, and the model was run 10 times with each different randomly re-assigned receptor map, and the simulation was repeated 10 more times for each re-assigned receptor map, for a total of 100 simulations each time. Figure 5 shows the results of randomly shuffling the empirical burden densities across the regions at the optimum point. This randomly reshuffled manipulation yields a significantly worse fit compared to the actual empirical burden densities (as shown by the Wilcoxon statistics in the boxplot). We additionally evaluate the quality of the simulation results with the optimized parameters with original (i.e., not shuffled) burdens and with the homogeneous BEI model. Finally, we examine the relevance of each type of burden by optimizing them in isolation from each other (i.e., zeroing the other one out), and comparing the results. The whole comparisons include both burdens in isolation, both burdens simultaneously, and with the homogeneous (i.e., BEI) model.

### Participants

Empirical data were obtained from the Alzheimer’s Disease Neuroimaging Initiative (ADNI) database (adni.loni.usc.edu), which is a longitudinal multi-site study designed to develop biomarkers for Alzheimer’s disease (AD) across all stages. The inclusion criteria for AD patients was the NINCDS-ADRDA criteria, which contains only clinical features [22], and an MMSE score below 24. For both HC and MCI, the inclusion criteria were a MMSE (Mini-Mental State Examination) score between 24 and 30, as well as age between 55 and 90 years. Also, for MCI, participants had to have a subjective memory complaint and abnormal results in another neuropsychological memory test. Imaging and biomarkers were not used for the diagnosis.

### Data acquisition and processing

All the data in this study were previously used in Stefanovski et al. [15] work, so we will present here an abridged version of the processing performed on the original data and refer to the original work for the details. All images used in this study were taken from ADNI-3, using data from Siemens scanners with a magnetic field strength of 3T.

#### Structural MRI

For each included participant, we created a brain parcellation for our structural data using FLAIR, following the minimal preprocessing pipeline [23] of the Human Connectome Project (HCP) using Freesurfer^1^ [24], FSL [25–27], and connectome workbench^2^. Therefore, we used T1 MPRAGE, FLAIR, and fieldmaps for the anatomical parcellation. We then registered the subject cortical surfaces to the parcellation of Glasser et al. [28] using the multimodal surface matching (MSM) tool [29]. In this parcellation, there were 379 regions: 180 left and 180 right cortical regions, 9 left and 9 right subcortical regions, and 1 brainstem region.

#### PET images

For A$\beta$, we used the version of AV-45 PET already preprocessed by ADNI, using a standard image with a resolution of 1.5mm cubic voxels and matrix size of $160 \times 160 \times 96$, normalized so that the average voxel intensity was 1 and smoothed out using a scanner-specific filter function. Then, a brainmask was generated from the structural preprocessing pipeline (HCP) and used to mask the PET image. We received in each voxel a relative A$\beta$ burden which is aggregated according to the parcellation used for our modeling approach. Subcortical region PET loads were defined as the average SUVR in subcortical gray matter (GM), normalized by the intensity of the cerebellum. With the help of the connectome workbench tool, using the pial and white matter surfaces as ribbon constraints, we mapped the Cortical GM PET intensities onto individual cortical surfaces. Finally, using the multimodal Glasser parcellation we derived average regional PET loads.

For tau, we also used ADNI’s preprocessed version of AV-1451 (Flortaucipir) following the same acquisition and processing, resulting in a single relative tau value for each voxel. Then, these values were also aggregated to the selected parcellation, following the already mentioned steps. The final average regional tau loads were obtained in the Glasser parcellation. Both mean burden values can be found, for each cohort, in Table 2.

#### DWI

Individual tractographies were computed only for included HC participants, and they were averaged to a standard brain template (see below). Preprocessing was mainly done with the MRtrix3 software package^3^.

In particular, the following steps were performed: First, we denoised the DWI data [30], followed by motion and eddy current correction^4^. Then, B1 field inhomogeneity correction (ANTS N4), followed by a brainmask estimation from the DWI images. Next, we normalized the DWI intensity for the group of participants, which was used to generate a WM response function [31], and created an average response function from all participants. Next, we estimated the fiber orientation distribution and the average response function [32] using the subject-normalized DWI image, to generate a five-tissue type image. Finally, we used the iFOD2 algorithm [33] and the SIFT2 algorithm [34] to get the weighted anatomical constrained tractography [35], to end up merging all information into the Glasser connectome, resulting in a structural connectome (SC).

The participating centers of ADNI are following centrally controlled protocols, which are described in detail at the ADNI website^5^. This includes the set-up of the MRI protocol by central ADNI institutions and quality checks with phantoms. However, there indeed remain differences between ADNI centers and ADNI phases. In the Supplemental material, we added a table with the DTI metadata of the 15 healthy controls that were used for structural connectivity calculation in this study (from [15]). Here, we see, despite the focus on only one scanner type from Siemens, that the data uses two different MRI protocols that only slightly differ: ADNI3 basic and ADNI3 advanced, which were designed to be compatible with each other^6^. The main difference remains in the TR and TE values, due to the different extent of measured sequences. A recent study showed for ADNI data including the used protocols that a cross-center harmonization indeed leads to significant differences in diffusion imaging derivatives, but the relationship with clinical data did not change, no matter if the harmonization was performed or not [36]. We, therefore, employed standardized preprocessing steps including quality controls, as outlined in the minimal processing pipeline of the Human Connectome Project [23]. To address the remaining effects, we have employed a methodological approach to compensate for this bias: we decided to use a group-based structural connectivity template for all participants. This comes with the benefit of standardizing the underlying network to an unaffected brain, which is then influenced by A$\beta$ and Tau. Even though the group-based template is derived from data from several ADNI study sites, this approach protects from introducing artificial differences between the subjects, as all are using the same structural connectivity. Also, it must be taken into account that we used an averaged SC from all healthy controls, thus further reducing any systematic bias of this kind.

#### fMRI

With respect to the processing of the fMRI data, the images were initially preprocessed in FSL FEAT and independent component analysis-based denoising (FSLFIX) following a basic pipeline [15]. Time courses for noise-labeled components, along with 24 head motion parameters, were then removed from the voxel-wise fMRI time series using ordinary least squares regression.

The resulting denoised functional data were spatially normalized to the MNI space using Advanced Normalization Tools (version 2.2.0). Mean time series for each parcellated region were then extracted, and interregional FC matrices were estimated using Pearson correlations between each pair of regional time series. Dynamic FC matrices were also calculated for the empirical data, as outlined below.

### Generation of a standard brain template

As previously done [15], we average the SCs of all HC participants, using an arithmetic mean

$$\begin{aligned} C_\mu = \left( \sum \limits _{i =1}^n C_i\right) /n = (C_1 + C_2 + . . . + C_n)/n \end{aligned}$$

wherein $C_\mu$ is the averaged SC matrix, *n* is the number of HC participants and $C_i$ is the individual SC matrix.

However, as matrices in this context are large (i.e., 379 regions), the average input to any given node can be too large for the DMF, making fitting and processing in general more difficult. Thus, we discarded the traditional normalization of dividing the matrix elements by their maximum, and used a slightly different approach, instead. First, we added one and applied the logarithm to every entry, as $lC = log(C_\mu +1)$. Then, we computed the maximum input any node could receive for a unitary unit input current, $maxNodeInput = max_j(\sum _i(lC_{i,j}))$, and finally, we normalized by $0.7 * lC / maxNodeInput$, where 0.7 was chosen to be a convenient normalization value. Observe that this constant is actually multiplying another constant *G* in the model which we fit to empirical data, so its actual value can safely be changed.

In Fig. 3, we can find the SC matrix and organization graph, where we can observe that the general characteristics of physiological SCs such as symmetry, laterality, homology, and subcortical hubs are maintained in the averaged connectome. The election of the averaged SC allowed us to control all factors (e.g., atrophy), which matched our objective of simulating the activity from both healthy and “pathogenic” modifications by A$\beta$ and tau.

### Balanced excitation-inhibition (BEI) model

In this work, we used the dynamic mean field (DMF) model proposed by Deco et al. [18], which consists of a network model to simulate spontaneous brain activity at the whole-brain level. Following the original formulation, each node represents a region of interest (i.e., a brain area) and the links represent the white matter connections between them. In turn, each node is a reduced representation of large ensembles of interconnected excitatory and inhibitory integrate-and-fire spiking neurons (as in the original, respectively 80% and 20% neurons), to a set of dynamical equations describing the activity of coupled excitatory (*E*) and inhibitory (*I*) pools of neurons, based on the original reduction of Wong and Wang [37]. In the DMF model, excitatory synaptic currents, *I*(*E*), are mediated by NMDA receptors, while inhibitory currents, *I*(*I*), are mediated by $GABA_A$ receptors. Both neuronal pools are reciprocally connected, and the inter-area interactions occur at the excitatory level only, scaled by the structural connectivity $C_{kj}$ (see the “Structural MRI” section).

To be more specific, the DMF model is expressed by the following system of coupled differential equations:

1 $$\begin{aligned} I_k^{(E)} = W_E\,I_o + w_+\,J_N \, S_k^{(E)} + J_N G \sum \limits _j C_{kj} S_j^{(E)} - J_k S_k^{(I)}+ I_{ext} \end{aligned}$$

2 $$\begin{aligned} I_k^{(I)} = W_I\,I_o + J_N S_k^{(E)} - S_k^{(I)} + \lambda J_N G \sum \limits _j C_{kj} S_j^{(E)} \end{aligned}$$

3 $$\begin{aligned} r_k^{(E)} = H^{(E)}\left( I_k^{(E)}\right) = \frac{M_k^E \left( a_E I_k^{(E)} - b_E\right) }{1 - \textrm{exp}\left( -d_E M_k^E \left( a_E I_k^{(E)} -b_E\right) \right) } \end{aligned}$$

4 $$\begin{aligned} r_k^{(I)} = H^{(I)}\left( I_k^{(I)}\right) = \frac{M_k^I \left( a_I I_k^{(I)} - b_I\right) }{1 - \textrm{exp}\left( -d_I M_k^I \left( a_I I_k^{(I)} -b_I\right) \right) } \end{aligned}$$

5 $$\begin{aligned} \dot{S}_k^{(E)} = -\frac{S_k^{(E)}}{\tau _E} + \left( 1 - S_k^{(E)}\right) \, \gamma H^{(E)}\left( I_k^{(E)}\right) \end{aligned}$$

6 $$\begin{aligned} \dot{S}_k^{(I)} = -\frac{S_k^{(I)}}{\tau _I} + H^{(I)}\left( I_k^{(I)}\right) \end{aligned}$$

Here, the last *two* equations should add, when integrating, an uncorrelated standard Gaussian noise term with an amplitude of $\sigma = 0.01nA$ (using Euler-Maruyama integration). In these equations, $\lambda$ is a parameter that can be equal to 1 or 0, indicating whether long-range feedforward inhibition is considered ($\lambda = 1$) or not ($\lambda = 0$). In the above equation, the kinetic parameters are $\gamma = 0.641/1000$ (the factor 1000 is for expressing everything in ms),  $\tau _E = \tau _{NMDA}$, and $\tau _I = \tau _{GABA}$. The excitatory synaptic coupling $J_{NMDA} = 0.15$ (nA). The overall effective external input is $I_0 = 0.382$ (nA) scaled by $W_E$ and $W_I$, for the excitatory pools and the inhibitory pools, respectively. The effective time constant of NMDA is $\tau _{NMDA}= 100$ ms [37]. The values of $W_I$, $I_0$, and $J_{NMDA}$ were chosen to obtain a low level of spontaneous activity for the isolated local area model. The values of the gating variables can be found in Table 1.

**Table 1:** Gating variables in the BEI model

Excitatory gating variables	Inhibitory gating variables
$a_E = 310$ ($nC^{-1}$)	$a_I= 615$ ($nC^{-1}$)
$b_E = 125$ (Hz)	$b_I = 177$ (Hz)
$d_E = 0.16$ (s)	$d_I = 0.087$ (s)
$\tau _E = \tau _{NMDA} = 100$ (ms)	$\tau _I = \tau _{GABA} = 10$ (ms)
$W_E = 1$	$W_I = 0.7$

As mentioned, the DMF model is derived from the original Wong and Wang model [37] to emulate resting-state conditions, such that each isolated node displays the typical noisy spontaneous activity with low firing rate ($H^{(E)}\sim 3Hz$) observed in electrophysiology experiments, reusing most of the parameter values defined there. We also implemented the feedback inhibition control (FIC) mechanism described by Deco et al. [18], where the inhibition weight, $J_n$, and was adjusted separately for each node *n* such that the firing rate of the excitatory pools $H^{(E)}$ remains clamped at 3 Hz even when receiving excitatory input from connected areas. Deco et al. [18] demonstrated that this mechanism leads to a better prediction of the resting-state FC and to a more realistic evoked activity. We refer to this model as the balanced excitation-inhibition (BEI) model. Although the local adjustments in this model introduce some degree of regional heterogeneity, the firing rates are constrained to be uniform across regions so we consider this BEI model as a homogeneous benchmark against which we evaluate more sophisticated models that allow A$\beta$ and tau to affect intrinsic dynamical properties across regions.

**Fig. 2:**
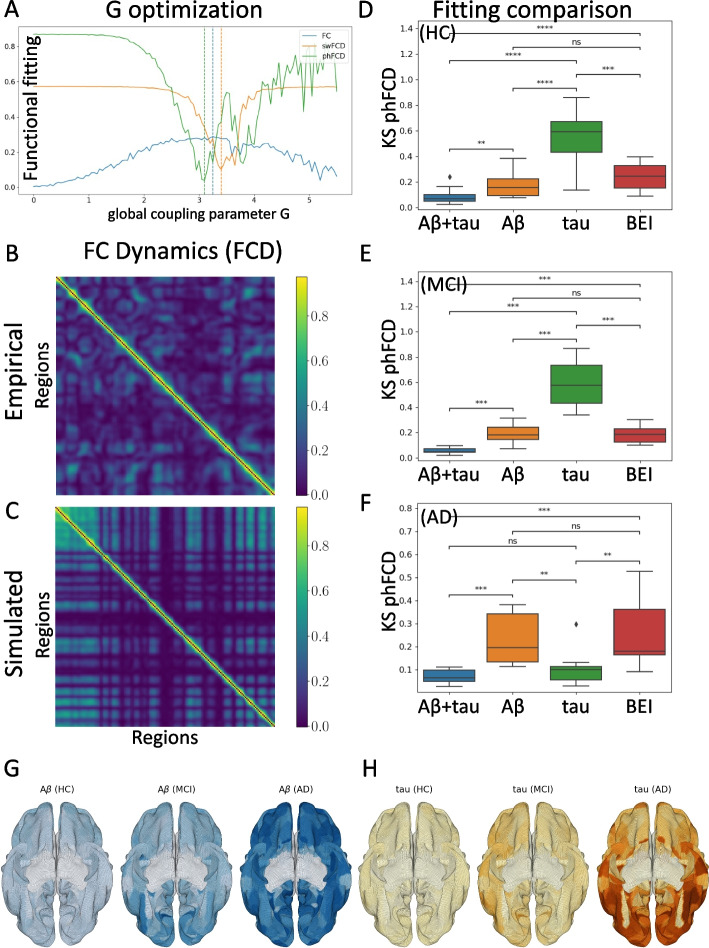
Optimization and evaluation of the model: First, using only HC subjects, the global coupling parameter *G* is found, and then the model is adjusted to minimize the distance between the empirical and simulated fMRI data, taking into account the regional burden distributions. **A** Minimization of *G* between 0 and 5.5, for functional connectivity (FC), sliding-window functional connectivity dynamics (swFCD), and phase FCD (phFCD). Given their strong similarity in the results, phFCD was used for all subsequent computations. **B**, **C** Shows the normalized (in [0, 1]) FCD distributions for the empirical data (top) and the simulated model at the optimal result (bottom). **D**, **E**, **F** Analysis of the impact (smaller values are better) of the different burdens with respect to their impact on the phFCD (KS distance) when optimized together and in isolation, with the homogeneous state as a reference. Clearly, in all cases, the combined burden outperforms any other model. However, as can be seen, the results for AD clearly show that tau alone accounts for the vast majority of the weight of the impact on brain activity (**F**), while for MCI patients it is A$\beta$ who dominates (**E**). For HC patients we also see a predominance of A$\beta$, although with less difference between the model incorporating A$\beta$ and tau vs. A$\beta$ in isolation (**D**). Average distributions of A$\beta$ (**G**) and tau burdens (**H**) over each cohort (using ADNI’s database). Colors correspond to the normalized burden of each protein. The increase in A$\beta$ and tau can be clearly seen

Following the Glasser parcellation [23], we considered $N = 379$ brain areas in our whole-brain network model. Each area *n* receives excitatory input from all structurally connected areas into its excitatory pool, weighted by the connectivity matrix, obtained from dMRI (see the “DWI” section). Furthermore, all inter-area E-to-E connections are equally scaled by a global coupling factor *G*. This global scaling factor is the only control parameter that is adjusted to move the system to its optimal working point, where the simulated activity maximally fits the empirical resting-state activity of healthy control participants. Simulations were run for a range of *G* between 0 and 5.5 with an increment of 0.05 and with a time step of 1 ms. For each *G*, we ran 200 simulations of 435 s each, in order to emulate the empirical resting-state scans from 17 participants. The optimum value found, for the *phFCD* observable, is for $G=3.1$. See Fig. 2A.

### Simulated BOLD signal

Once we have obtained the simulated mean field activity, we need to transform it into a BOLD signal we used the generalized hemodynamic model of Stephan et al. [38]. We compute the BOLD signal in the *k*-th brain area from the firing rate of the excitatory pools $H^{(E)}$, such that an increase in the firing rate causes an increase in a vasodilatory signal, $s_k$, that is subject to auto-regulatory feedback. Blood inflow $f_k$ responds in proportion to this signal inducing changes in blood volume $v_k$ and deoxyhemoglobin content $q_k$. The equations relating these biophysical variables are:

7 $$\begin{aligned} \frac{d s_k}{dt}{} & {} = 0.5 r_k^{(E)} + 3 - k s_k - \gamma (f_k-1) \nonumber \\ \frac{d f_k}{dt}{} & {} = s_k \nonumber \\ \tau \frac{d v_k}{dt}{} & {} = f_k - v_k^{\alpha ^{-1}} \\ \tau \frac{d q_k}{dt}{} & {} = f_k \frac{1-(1-\rho )^{f_k^{-1}}}{\rho } - q_k \frac{v_k^{\alpha ^{-1}}}{v_k}\nonumber \end{aligned}$$

with finally

$$\begin{aligned} B_k = v_0 \left[ k_1(1-q_k)+k_2\left( 1-\frac{q_k}{v_k}\right) +k_3(1-v_k) \right] \end{aligned}$$

being the final measured BOLD signal.

We actually used the updated version described later on [38], which consists on introducing the change of variables $\hat{z} = ln z$, which induces the following change for $z=f_k$, $v_k$ and $q_k$, with its corresponding state equation $\frac{dz}{dt} = F(z)$, as:

$$\begin{aligned} \frac{d\hat{z}}{dt} = \frac{d\;ln(z)}{dz} \frac{dz}{dt} = \frac{F(z)}{z} \end{aligned}$$

which results in $z(t)=exp(\hat{z}(t))$ always being positive, which guarantees a proper support for these non-negative states, and thus numerical stability when evaluating the state equations during evaluation.

### A$\beta$-Tau model

In our heterogeneous model, A$\beta$ and Tau are introduced, at the formulae for the neuronal response functions, $H^{(E,I)}$ (excitatory/inhibitory), into the gain factor $M_k^{(E,I)}$ for the *k*-th area as

8 $$\begin{aligned} M_k^E = \left( 1 + b^E_{A\beta } + s^E_{A\beta } A\beta _k\right) \left( 1 + b^E_\tau + s^E_\tau tau_k\right) \end{aligned}$$

9 $$\begin{aligned} M_k^I = \left( 1 + b^I_{A\beta } + s^I_{A\beta } A\beta _k\right) \left( 1 + b^I_\tau + s^I_\tau tau_k\right) \end{aligned}$$

where $b^{(E,I)}_{(A\beta ,\tau )}$ are the excitatory/inhibitory A$\beta$ and tau bias parameters, while $s^{(E,I)}_{(A\beta ,\tau )}$ are the respective scaling factors. These are the 8 (from which actually only 6 are needed as tau only affects excitatory neurons [39], see next section) parameters that we will optimize for each subject individually.

### Constraints

Based on previous neuroscientific experiments [4], we included constraints on the direction of effect of A$\beta$ and tau (i.e., inhibitory vs. excitatory influence). We introduced the following constraints:

- A$\beta$ produces inhibitory GABAergic interneuron dysfunction [6, 40], thus we can infer that $s^I_{A\beta } < 0$.
- A$\beta$ produces impaired glutamate reuptake [6, 40], so we can introduce the bound $s^E_{A\beta } > 0$.
- Tau appears to target excitatory neurons [39], so we can safely consider that $b^I_\tau = s^I_\tau = 0$.
- Tau binds to synaptogyrin-3, reducing excitatory synaptic neurotransmitter release [41], thus $s^E_\tau < 0$.

Although the interplay between A$\beta$ and tau is not completely known [4], but there is evidence that A$\beta$ promotes tau by cross-seeding [42, 43], thus the cross-term factors (i.e., the ones resulting from the multiplication of A$\beta$ and tau scaling parameters) play a crucial role to elucidate the final impact of the combined burden.

### Observables

#### Edge-centric FC

**Fig. 3:**
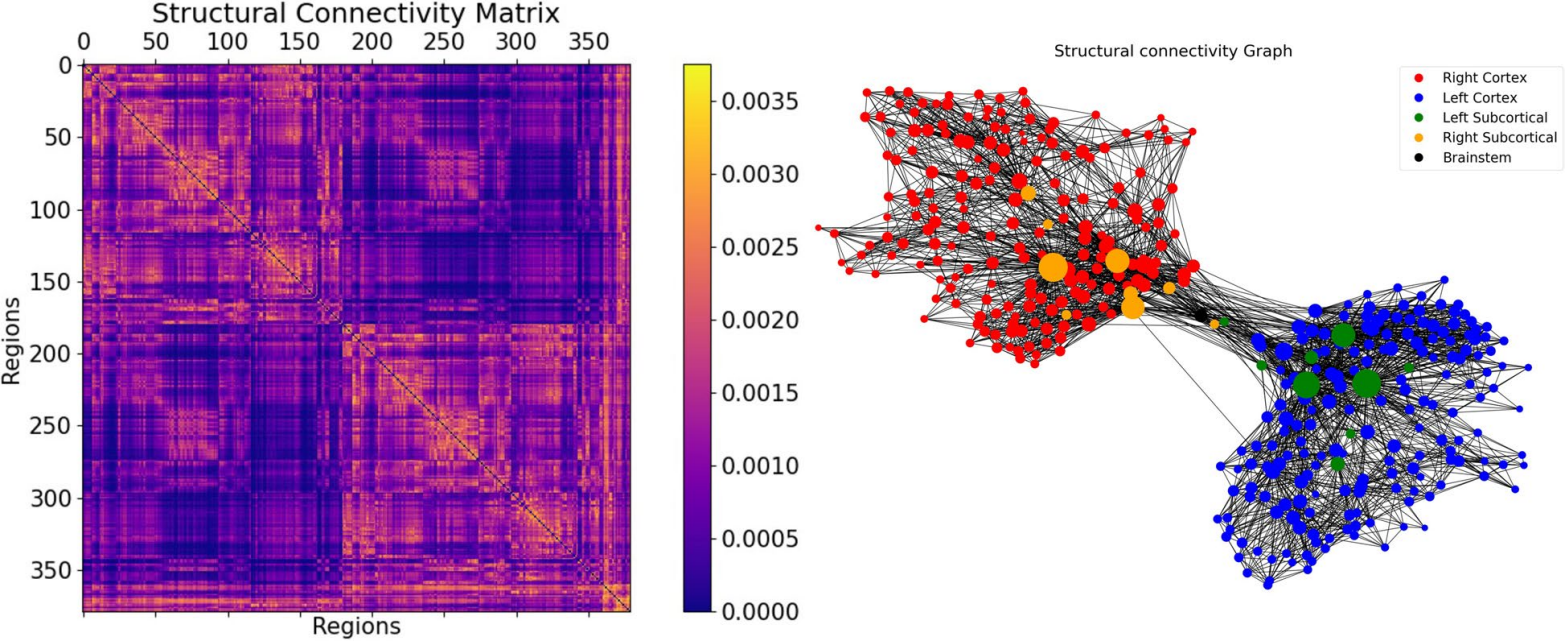
Visualization of the SC graph, in matrix form (left) and as a graph showing the strongest 5% of connections. Node positions are computed with Fruchterman and Reingold’s [44] algorithm, which assumes stronger forces between tightly connected nodes. Besides the high degree of symmetry, we can observe the laterality is kept in the graph structure (also for subcortical regions). Node size linearly represents the graph theoretical measure of structural degree for each node. As we can see, the most important hubs are in the subcortical regions

The static edge-level FC is defined as the $N\times N$ matrix of BOLD signal correlations between brain areas computed over the entire recording period (see Fig. 3). We computed the empirical FC for each human participant and for each simulated trial, as well as for the group-averages SC matrix of the healthy cohort. All empirical and simulated FC matrices were compared by computing the Pearson correlation between their upper triangular elements (given that the FC matrices are symmetric).

#### swFCD

The most common and straightforward approach to investigate the temporal evolution of FC is the sliding-window FC dynamics (swFCD) [45]. This is achieved by calculating the correlation matrix, *FC*(*t*), restricted to a given time-window $(t-x:t+x)$, and successively shifting this window in time resulting in a time-varying $FC_{NxNxT}$ matrix (where *N* is the number of brain areas and *T* the number of time windows considered). Here, we computed the FC over a sliding window of 30 TRs (corresponding approximately to 1.5 min) with incremental shifts of 3 TRs. This FCD matrix is defined so that each entry, ($FCD(t_x,t_y)$) corresponds to the correlation between the FC centered at times $t_x$ and the FC centered at $t_y$. In order to compare quantitatively the spatio-temporal dynamical characteristics between empirical data and model simulations, we generate the distributions of the upper triangular elements of the FCD matrices over all participants as well as of the FCD matrices obtained from all simulated trials for a given parameter setting. The similarity between the empirical and model FCD distributions is then compared using the KS distance, $D_{KS}$, allowing for a meaningful evaluation of model performance in predicting the changes observed in dynamic resting-state FC. However, the fundamental nature of the swFCD technique implies the choice of a fixed window length, which limits the analysis to the frequency range below the window period, so the ideal window length to use remains under debate [46].

#### phFCD

In an attempt to overcome the limitations of the sliding-window analysis, a few methods were proposed to estimate the *FC*(*t*) at the instantaneous level. For instance, phase functional connectivity dynamics (*phFCD*) consists in computing the phase coherence between time series at each recording frame [21]. In brief, the instantaneous BOLD phase of area *n* at time *t*, $\theta _n(t)$, is estimated using the Hilbert transform. Given the phase, the angle between two BOLD signals is given by their absolute phase difference: $\Theta _{np}=|\theta _n(t)-\theta _p(t)|$. Then, the *phFCD*(*t*) between a pair of brain areas *n* and *p* is calculated as:

$$\begin{aligned} phFCD{np}(t) = cos(\Theta _{np}(t)), n, p \in N=1, ..., N \end{aligned}$$

with *N* the number of brain regions considered in the parcellation used. To compare two phFCD matrices among themselves, e.g., a simulated and an empirical one, again the KS distance is usually used.

### Full optimization

To efficiently optimize the 6-dimensional function described before for the three bias and scaling values, a simple local optimization-based approach such as conjugate gradients cannot be used, as this is a (usually) ill-posed problem with a global minimum surrounded by many local minima. Instead, we need to resort to a global optimization algorithm. In our case, we used a Bayesian minimization algorithm using Gaussian Processes (GP), which approximates the function using a multivariate Gaussian. In particular, our implementation uses the *gp_minimize* method from the *scikit-optimize* Python library^7^. At its core, the method approximates the objective function with a Gaussian process, assuming that the values follow a multivariate Gaussian. The covariance of the function values is given by a GP kernel between the parameters. With this information, the algorithm chooses the next parameter to evaluate by selecting the acquisition function over the Gaussian prior. The error measure used was the KS distance between the empirical BOLD signal and the average over a number of trials (10 in our case) of the simulated signal, and we let the function run for 100 iterations. In all cases, the results moved significantly away from the priors.

## Results

We used diffusion MRI to generate the structural connectomes of 17 healthy control (HC) subjects, 9 subjects with mild cognitive impairment (MCI), and 10 subjects with Alzheimer’s disease (AD) from ADNI. These subjects were also analyzed in the studies by Stefanovski et al. [15] and Triebkorn et al. [16]. For the description of subject characteristics, see Table 2 and Fig. 3.

To ensure a sufficient sample size for our computationally expensive analysis, we used the G$*$Power [47] software to conduct statistical power calculations based on a two-group Wilcoxon-Mann-Whitney test, with a significance level $\alpha =0.05$ and power $1-\beta = 0.8$. Assuming a standard deviation $\sigma = 0.05$ (a reasonable assumption given our results below), we calculated that the minimum effect size in this setting would be $d = 1.1$, which implies that the minimum detectable difference between the means of the control population and any of the other two would be around 0.055.

**Table 2:** Epidemiological information of the population used in this study

Diagnosis	*n* (female)	Mean age	$\sigma$	Min. age	Max. age	Mean MMSE	$\sigma _{MMSE}$	Min. MMSE	Max. MMSE	Mean A$\beta$	Mean tau
HC	17 (10)	70.8	4.3	63.1	78.0	29.3	0.7	28	30	1.31	1.53
MCI	9 (3)	68.8	5.8	57.8	76.6	27.4	1.5	25	30	1.52	1.80
AD	10 (5)	72.0	9.6	55.9	86.1	21.3	6.8	9	30	2.01	2.46

### Fitting the homogeneous model

As a first step, we evaluated the capability of the homogeneous BEI model to reproduce the empirical properties of resting-state FC data. To this end, we fitted the global coupling parameter, *G*, without considering heterogeneity by setting all regional gain parameters $M_{(E,I)}=1$ [18]. Then, we evaluated the ability of the model to reproduce three different properties of empirical resting-state fMRI recordings: edge-level static FC, swFCD, and phFCD (see the “Methods” section for further details.) The results of this analysis are shown in Fig. 2A. To remove differences across subjects related to age, we considered averaged values across subjects over the healthy control group and took an equivalent number of simulated trials with the same duration as the human experiments (see the “Methods” section). Following a previous research [19], we focused on fitting the phFCD, as it better captures the spatiotemporal structure of the fMRI data, thus being a stronger constraint on the model. Indeed, where FC fits are consistently high across a broad range of *G* values, phFCD yields a clear global optimum at $G=3.1$. Thus, we choose to use phFCD for all further fitting procedures.

### Introducing A$\beta$ and tau heterogeneity

Once the global coupling parameter was found, we introduced the regional heterogeneity in the distributions of A$\beta$ and tau, and studied how their introduction lead to a better representation of neural dynamics, i.e., improved the fitting of phFCD. Spatial maps for average values of each form of protein burden used in our modeling are shown in Fig. 2G (for A$\beta$) and H (for tau) for each cohort. Also, in the Supplemental material, we present a graph with each of these burdens for each cohort in the MMSE classification. For some individuals, (mainly HC subjects, e.g., as subject 003_S_6067 in the ADNI database, with $\rho =0.92$, $p<0.001$) the A$\beta$ and tau distributions were strongly correlated, while for others the two maps showed a weaker correlation (e.g., subject 036_S_4430, with $\rho =0.10$, $p=0.04$.) This observation indicates that each protein burden introduces a different form of biological heterogeneity to the benchmark BEI model, and thus should be modeled separately in our simulations.

We introduced these types of heterogeneity by modulating the regional gain functions $M_{(E,I)}$ at the optimal working point of the homogeneous BEI model found at the previous stage ($G=3.1$), through the bias and scaling parameters introduced above, denoted $b^E_{A\beta }$ and $s^E_{A\beta }$ for A$\beta$, and $b^E_\tau$ and $s^E_\tau$ for tau, all for the excitatory populations, and similarly for the inhibitory populations with superscript *I*. We performed a search in parameter space with constraints introduced from experimental observations (see the “Constraints” section), to find the optimal working point for the two protein burdens simultaneously. This results in an 8-degree of freedom optimization, which is reduced to six degrees due to the constraints. For the optimization, we used a Bayesian optimization algorithm using Gaussian processes (see the “Full optimization” section). We also performed a simplified search, limited to the two-variable $b^I_{A\beta }$ and $s^I_{A\beta }$ space, i.e., the inhibitory bias and scaling of the A$\beta$ influence on inhibitory neuron parameters (Eq. 9). In this case, the 2D optimization results showed a decrease in the neuronal activity with increasing A$\beta$ concentration, confirming previous results [15]. On average, for each group of subjects, we got the results shown numerically in Table 3.

**Table 3:** Resulting averaged parameters from the optimization procedure. In parenthesis, the respective standard deviations

Cohort	$b^E_{A\beta }$	$s^E_{A\beta }$	$b^E_\tau$	$s^E_\tau$	$b^I_{A\beta }$	$s^I_{A\beta }$
AD	0.2 (0.5)	2.3 (1.2)	− 0.4 (0.6)	− 2.6 (0.8)	0.2 (0.6)	− 2.5 (0.8)
MCI	0.4 (0.7)	1.7 (1.5)	− 0.5 (0.5)	− 2.8 (0.7)	− 0.1 (0.8)	− 2.1 (1.2)
HC	0.1 (0.8)	1.7 (0.9)	− 0.5 (0.6)	− 2.8 (1.0)	0.3 (0.6)	− 3.1 (1.0)

**Fig. 4:**
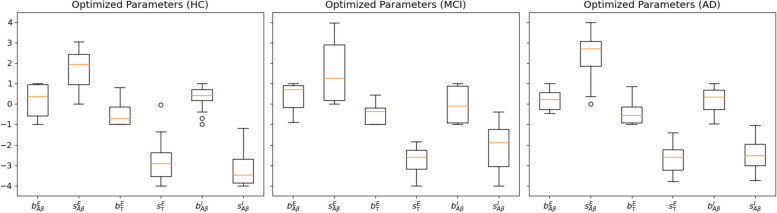
Parameter values found after the optimization stage for HC, MCI and AD subjects. Observe that all $b^{(E,I)}_{(A\beta ,\tau )}$, the excitatory/inhibitory A$\beta$ and tau bias parameters, have negligible values, while the scaling parameters $s^{(E,I)}_{(A\beta ,\tau )}$ present non-null values. Of note, the *p*-values between the different scaling parameters across the cohorts are different in a moderately significant way ($p<0.03$), remarkably between HC and AD, but usually not between MCI and AD. In these plots, boxes extend from the lower to upper quartile values of the data, adding an orange line at the median. Also, whiskers are used to show the range of the data, extending from the box

The results of the fitting can be seen visually in Fig. 4. This figure shows that there is a clear regime in which all three empirical properties are fitted well by the model, particularly for the values shown above, where a fitting of phFCD of 0.13 is achieved for the AD subjects, while the reference homogeneous value is equal to 0.5.

We repeated the analysis based on the AT(N) classification [48–51], now grouping the subjects into four categories: A−T−, A+T−, A−T+, and A+T+, according to their average A$\beta$ and tau SUVR levels. As thresholds, we used 0.9 of the average SUVr value for each burden, which results in a threshold of 1.4219 for A$\beta$, and 1.67 for tau. As we have A$\beta$ PET and Tau PET available, we were able to assign the A and T dimensions of AT(N), as defined in [2]. As a result, we redistributed our original cohorts into four sets, each with a different number of subjects: A−T−: 15, A−T+: 4, A+T−: 5, and A+T+: 13. As the number for A−T+ and A+T− cohorts were small, we expected to get inconclusive results when comparing with these two cohorts. The results show very similar behaviors for the A-T- group with respect to the HC group and for the A+T+ with respect to the AD one. However, for the other two groups, given the low number of subjects, the results are mixed or inconclusive. A detailed explanation can be found in the companion Supplemental material.

### Analysis of burden impact

**Fig. 5:**
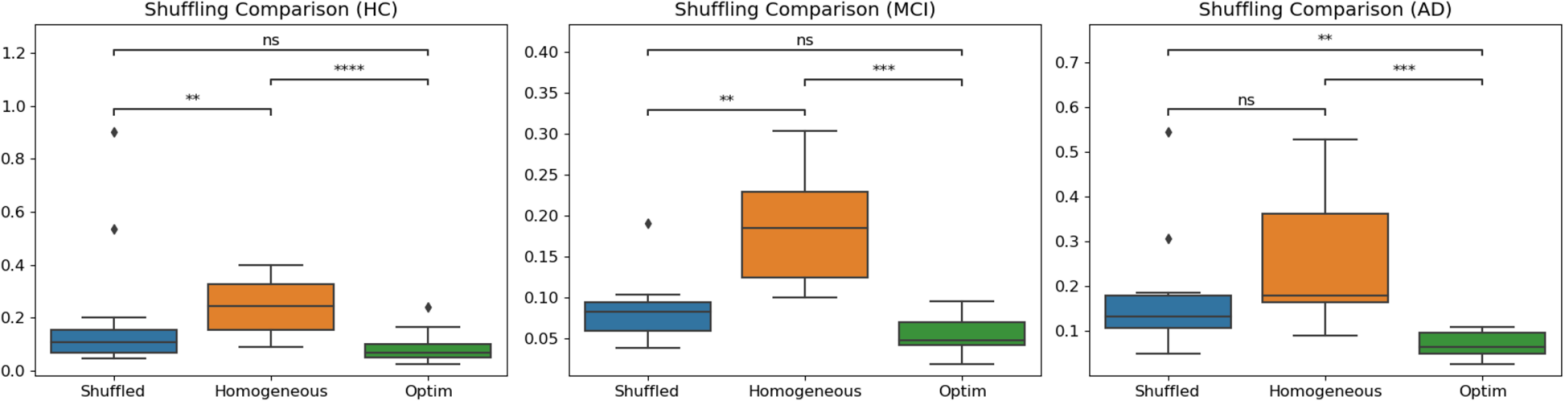
Comparison between the homogeneous model, the optimum result obtained with the heterogeneous model (optim), and the same parameter values but with shuffled burdens. As can be seen, the differences in fit statistics between models are significant. In particular, for the AD cohort, the median phFCD correlation between model and data showed $r<0.1$ for the heterogeneous model, and $r\approx 0.2$ for the BEI model. In all subject groups, the difference between these two models is clear, with $p<0.0005$

For the optimal parameter values resulting from model fitting, we simulated each dynamical model 10 times for each subject to account for the inherently stochastic nature of the models and computed the respective measures of model fit. Figure 5 shows the distributions of fit statistics across runs for the homogeneous and the heterogeneous models for the different cohorts. In addition, we show results for a null ensemble of models, in which the regional burden values were spatially shuffled to generate surrogates with the same spatial autocorrelation as the empirical data. Across the benchmark property to which the data were fitted (phFCD), the models taking into account the regional burden heterogeneity perform better than the homogeneous model ($p < .0005$). We also found a consistent gradient of performance across all benchmarks, with the heterogeneous model performing best, and the homogeneous model showing the poorest performance. For each benchmark metric, the performance of the heterogeneous model was better than all other models (in all cases $p < .06$). Also, it must be noted that the differences in fit statistics between models are significant, as shown in Fig. 5. For example, for the AD cohort, the correlation of the median phase FCD between the fitted model and empirical data shows $r<0.1$ for the heterogeneous model, and $r\approx 0.2$ for the BEI model. In all subject groups, the difference between these two models is clear, with $p<0.0005$. In all reported results we used a Mann-Whitney-Wilcoxon test two-sided with Benjamini-Hochberg correction (*p*-value annotation legend: ns: $p <= 1.00e+00$, *: $1.00e-02< p <= 5.00e-02$, **: $1.00e-03< p <= 1.00e-02$, ***: $1.00e-04< p <= 1.00e-03$, ****: $p <= 1.00e-04$).

Finally, we performed an analysis comparing the impact of each type of burden, in isolation or together, onto the simulation results. In Figs. 2D-F we can see these results for the different cohorts, for A$\beta$ and tau, A$\beta$ alone, tau alone and the homogeneous BEI model, added for reference. As we can see, with respect to the homogeneous model, the best performance is systematically obtained by the combined action of both A$\beta$ and tau, giving a value with $p < 0.0004$ in all cases. However, for each cohort, each protein is shown to play a different role in the development of the disease. For AD subjects, the effect of A$\beta$ on the optimal combined result is small, with a $p < 0.0005$, while the influence of tau alone has a *p* value that does not allow us to distinguish between its effect and the combined effect of both proteins ($p=0.172$), implying a clear dominance of tau over A$\beta$ in this stage of the disease. Also, with respect to the homogeneous BEI model, tau presents $p < 0.005$, while A$\beta$ alone shows a much higher value ($p=0.339$), not allowing us to clearly distinguish between these two models. In the case of the MCI cohort, in Fig. 2E, we can observe that the effect of A$\beta$ alone clearly gives the major contribution to the final combined fitting, rather than tau, with a $p<0.0003$ between all cases. Finally, in the HC case in Fig. 2D, the effects of the A$\beta$ and tau proteins are close to the homogeneous BEI model, with A$\beta$ presenting a somewhat higher prevalence than tau. However, it is noticeable that the differences between this case and the previous one are small, showing that A$\beta$ already plays an important role even in HC subjects.

All the results just described can be put in the context of the minimum detectable difference, with a value of 0.055, which is the result of the statistical power calculations taking into account our cohort size, as described above. The results in Fig. 2D–E can be assessed in light of this result by observing that, in most of the cases, the difference between the average values is larger than the calculated minimum difference. For instance, for AD, the difference between the result computed for the combined A$\beta$ and tau burden, and A$\beta$ alone is 0.16, above our criterion. However, in this case, the difference of the combined burden with tau alone is 0.03, below our threshold, thus leading to a statistically non-significant assessment. This same analysis can be reproduced for each of the other cases, clearly explaining the significance of most of the results obtained, and showing that our cohort size is sufficient for this analysis.

### 2D A$\beta$ optimization

**Fig. 6:**
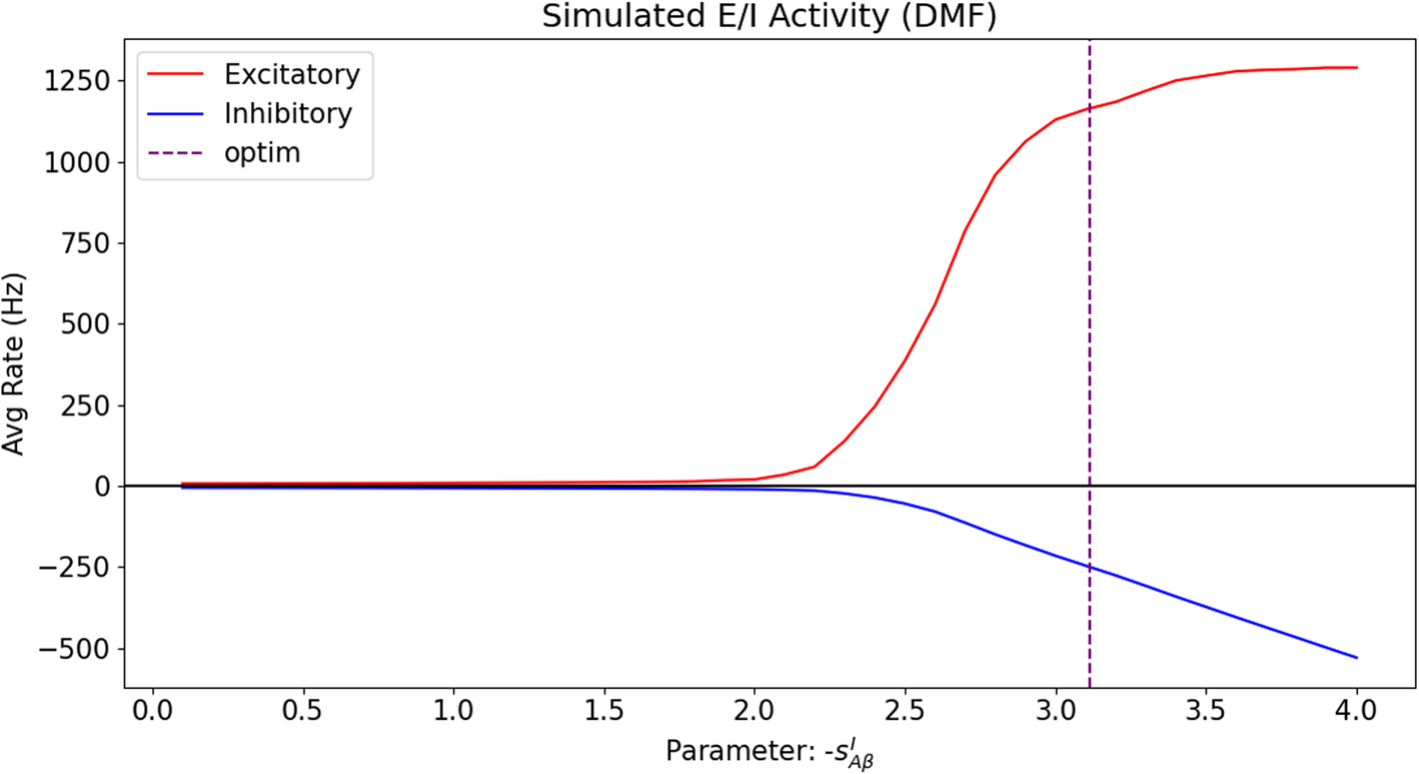
Excitatory and inhibitory mean firing rates as a function of the A$\beta$ inhibitory scaling $s^I_{A\beta }$, with all the other parameters of the model at the (averaged) fitted optimum values. For the purpose of clarity, the horizontal axis for the scaling has been taken as absolute values, to illustrate the behavior with increasing A$\beta$ loads. The vertical axis shows the firing rates of both excitatory and inhibitory populations. It can be clearly seen that the net effect of the burden is to increase the overall region firing rate, measured at the excitatory population. For the sake of clarity, the inhibitory firing rate has been vertically inverted (negated) to show their decreased effect on the excitatory population, thus confirming previous findings [15]. The vertical discontinuous line shows the optimum found for $s^I_{A\beta }$

We used our model to verify the results by Stefanovski et al. [15] by limiting our analysis to the parameters of A$\beta$ at the inhibitory level (i.e., the inhibitory bias $b^I_{A\beta }$ and scaling $s^I_{A\beta }$ parameters only, defined in Eq. 9). This way, we replicated the results from that study, even when using a different model (BEI model instead of the Jansen-Rit model [52]); a different expression for the burden, i.e., a linear approximation instead of a sigmoid; different units, etc. (See Fig. 4.) By analyzing the obtained data at the optimal fit, the same behavior of decreasing the neuronal activity of inhibitory neurons with the scaling parameter $s^I_{A\beta }$, corresponding to an increase in A$\beta$ concentration, is observed, as shown in Fig. 6.

## Discussion

In this paper, we studied the influence of the regional variability of two pathological proteins, namely A$\beta$ and tau, on cortical activity and E/I balance in the context of AD. We used whole-brain dynamic modeling, which allowed us to disentangle the separate and synergistic effects of these two proteins in silico. The incorporation of such heterogeneous patterns of neuropathology into whole-brain models of neuronal dynamics has been made possible by the availability of in-vivo quantitative PET imaging. We have shown that the heterogeneous model, which incorporates regional information on both types of neuropathological burdens more faithfully reproduces empirical properties of dynamic brain activity than the model with fixed and homogeneous parameters. Our findings highlight the central role of both types of burden in disturbing the E/I balance, supporting the hypothesis of hyperexcitation in AD. Regarding the individual influence of A$\beta$ and tau on brain activity, our results have shown a dominance of A$\beta$ influence on neural dynamics in earlier stages of AD (i.e., MCI) and even in healthy controls, while tau plays a larger role in later stages. These key findings highlight the prominent role of these pathological proteins in contributing to the abnormal brain activity patterns in the course of AD [53].

### How does burden heterogeneity shape neuronal dynamics?

We introduced burden heterogeneity into our dynamical model by modifying the regional excitability of neural population activity. We achieved this by modifying each brain region’s gain response function $M_i$ of inhibitory and excitatory populations, i.e., the net excitability of the according population. This was done in accordance with previous works exploring the effect of regional parameters on E/I balance [19], thus focusing on how the interaction of neuronal populations contributes to neuronal dynamics (i.e., FC or FCD). Our approach is different from the work by Stefanovski et al. [15], where the A$\beta$ burden was used to modulate regional E/I balance by negatively modulating the inhibitory time constant, slowing down synaptic transmission and thus increasing excitatory activity and producing a higher output of the pyramidal cell populations, resulting in a local hyperexcitation with high A$\beta$ loads. However, our approach, when limited to the effect of A$\beta$ in the early stages of the disease, results in the same behavior of neural populations as a function of A$\beta$, similarly resulting in a net increase of the excitatory activity with increased A$\beta$ burden. There are other approaches available to introduce heterogeneity, such as an adjustment of the inter-node connectivity to fit empirical and simulated FCs [54]; or variations of within- and inter-area connectivity [55]. However, based on the empirical evidence that the interplay of both burdens, A$\beta$ and tau, severely disrupt normal neuronal function, we decided to model their direct effect on the E/I balance.

In this paper, we have chosen to incorporate heterogeneity into the model by modulating population gain response functions $H^{(E,I)}$. Here, adjusting the gain function parameter $M_i$ allows us to demonstrate how local variations in the E/I balance will affect the net excitability of the population. We thus assume that changes in regional gain are the common final pathways of different neuropathology-related pathomechanisms. These mechanisms might have an influence on neuronal populations, i.e., result in realistic representation of direct effects of A$\beta$ and tau and also associated processes (i.e., non-direct effects).

In particular, we introduced regional variations of $M_i$ as the product of linear terms consisting of a constant (bias), and a scaling factor. This introduced eight degrees of freedom, which we could narrow down to six degrees of freedom by introducing constraints to the direction of effect based on previous research [4]. In sum, our model was created based on assumptions that A$\beta$ leads to GABAergic interneuron dysfunction and impaired glutamate reuptake, while tau leads to reduced synaptic neurotransmitter release in excitatory cells. This hypothesis-driven amount of degrees is substantially less than used in other models [54, 55], making a fast parameter optimization feasible, while ensuring sufficient biological realism. Furthermore, in all cases, the bias parameters for the different burdens (Fig. 4) were approximately 0, thus indicating that the influence of the bias parameters with respect to the homogeneous model can be ignored, further reducing computational complexity. The respective scaling parameters take non-negligible values, showing a linear relationship between A$\beta$ and tau on neural dynamics. We used Bayesian optimization using Gaussian Processes (see Methods) to address the challenge of multiple local minima that could trap traditional optimization methods.

### Evaluating A$\beta$ and tau impact

A large body of scientific literature focused on linking global and local brain dynamics to individual differences in cognitive performance scores [12] and showed that patients with AD and MCI show less variation in neuronal connectivity during resting-state, and even presented benchmarks for predictive models based on resting-state fMRI, revealing biomarkers of individual psychological or clinical traits [13]. However, the pattern of neuronal connectivity alterations has been incompletely understood. More recent work focused on the effect of A$\beta$ on hyperexcitability, and how A$\beta$ modulates regional E/I balance, resulting in local hyperexcitation in brain regions with high loads of A$\beta$ [15]. To our knowledge, no prior study has evaluated both types of neuropathological burdens, A$\beta$ and tau, simultaneously, linking neuropathological data with dynamic whole-brain modeling.

As explained in the Methods section, we compared the impact of each type of burden, in isolation or interaction, onto neural dynamics. We found that the model fitting optimum is systematically obtained by the interaction of both burdens, underlining the interaction of both proteins in disturbing neural activity. Also, we have found that for each condition (i.e., HC, MCI, or AD), each protein has a different impact on the brain dynamics. In the case of AD, A$\beta$ has a small impact on the combined result, while tau alone had almost all of the impact, showing its dominance over A$\beta$ in regard to generating abnormal brain dynamics. Also, in comparison to the homogeneous BEI model, we observed that tau is clearly distinguishable, but A$\beta$ is not. Taken together, these results imply that we cannot distinguish between the effect on the brain activity of both proteins together vs. the effect of tau alone, while the effect of A$\beta$ is clearly distinguishable from the combined effect. As a consequence, this allows us to conclude that the impact of tau in the late stage of the disease (AD) is clearly dominant over A$\beta$. In contrast, in MCI, the influence of A$\beta$ alone is clearly dominant over tau, see Fig. 2E. Finally, when studying the effect of both proteins in HC, we can observe that the effect of the A$\beta$ and tau proteins is close to the homogeneous BEI model, with A$\beta$ presenting a higher influence than tau. The influence of A$\beta$ both in MCI patients as well as in HC shows that A$\beta$ leads to a measurable change in brain dynamics in elderly people, independent of existing cognitive impairment. However, we acknowledge that on a pathophysiological level, there is a strong interplay between A$\beta$ and tau, and further (causal) research is needed to clearly discern the role each protein plays in the generation of neuronal dysfunction. Despite our findings from model fitting, we acknowledge that we only observe the current influence of A$\beta$ vs. tau in different disease stages in a cross-sectional cohort. Longitudinal examinations might also replicate the abundant evidence in the literature [4] that both proteins interplay a toxic feedback loop, which is ultimately responsible (perhaps among other factors) for the development of the disease.

Our analysis furthermore shows that edge-level measures of static FC offer loose constraints for model optimization, showing comparably high fit statistics across a broad range of values of the global coupling parameter. In contrast, fitting to dynamical functional connectivity shows a clear optimum, mirroring similar results reported previously [19]. We can conclude that fitting models to both static and dynamic properties is thus important for identifying an appropriate working point for each model.

Across all these properties, we observe that the model that incorporates the heterogeneous burden loads provides a better match to the data than the homogeneous BEI model, which does not incorporate a fitting of the gain response function of inhibitory and excitatory populations to the data. This shows that constraining regional heterogeneity by the protein burdens yields a more faithful replication of brain dynamics, as measured by empirical phFCD. The superiority of our model using heterogeneous, empirically estimated parameters, suggests that regional heterogeneity plays a significant role in shaping the effects of Alzheimer’s disease on spontaneous BOLD dynamics. As we already mentioned, it must be noted that the differences in fit statistics between models are significant. These results suggest that these empirical fit statistics have a good capacity to tease apart dynamical differences between models, which gives the opportunity to disentangle the influence of different pathomechanisms in vivo.

## Limitations

In our implementation, we used SC matrices derived from DWI. However, many factors such as myelination and diameter impact the conductivity of white matter tracts. Thus, assuming that coupling between different brain areas is not affected by this it may be a confounding factor.

On the other hand, DWI tractography already introduces heterogeneities in the form of different connections between nodes, representing the actual physical connections between them. However, given the nature of these measures, it assumes that all connections have the same conductivity in the fiber tracts. This is not completely realistic and it has been shown [56, 57] that adding these additional degrees of freedom improves substantially the result. Effective connectivity is usually defined as a causal connectivity measure, meaning the directional influences of one brain area or neural element over another [56, 58]. An effective connectivity measure would not only result in a much better fitting but also would allow us to discern more precisely the damaged areas in the brain. We hypothesize that such areas would show decreased effective connectivity on a global level for slow waves, and thus a decreased propagation of information, as a direct effect of the damage produced by the different burdens. However, computing effective connectivity is a complex process that we left as an avenue for future work.

It is important to mention that we included sub-cortical regions, which are particularly susceptible to off-target binding of the AV-1451 tracer, which may introduce a potential confound. For this study, we did not control the images for off-target binding. Recent studies show that, besides Tau tangles, AV-1451 also binds to neuromelanin, melanin, and other blood products [59]. This is a genuine restriction of the PET imaging method and we are not aware of a standard that corrects this phenomenon. Moreover, there is some controversy about whether this is always off-target binding or detecting tangles that other methods are not aware of [60]. In any case, it can be argued that the typical finding of this off-target binding affects AD, MCI, and controls to the same amount.

## Conclusion

In summary, we have presented a whole-brain dynamic model connecting the main protein burdens, namely A$\beta$ and tau, in different stages of AD and in HC. Our results not only reproduce previous research regarding E/I imbalance in AD, but also shed further light on the relative impact of each type of burden during different disease stages, opening new avenues to focus research efforts. As a general conclusion, our study shows that theory-driven whole-brain modeling enables us to do research on disease mechanisms in silico and to empirically compare competing hypotheses against each other and thus complements data-driven modeling such as machine learning. Thus, whole-brain modeling can incorporate sufficient biological realism to contribute to improved diagnostic procedures (i.e., enable the use of fMRI for diagnosis) and to discover new therapies (e.g., by simulating novel treatments).

### Supplementary Information

**Additional file 1.**

## Data Availability

All code for implementing computational models and reproducing our results will be available at the first author’s repository: https://github.com/dagush/WholeBrain/tree/master/Projects/AD-ABeta_and_Tau Data used in the preparation of this article were obtained from the Alzheimer’s Disease Neuroimaging Initiative (ADNI) database (adni.loni.usc.edu). As such, the investigators within the ADNI contributed to the design and implementation of ADNI and/or provided data but did not participate in the analysis or writing of this report. A complete listing of ADNI investigators can be found at: https://adni.loni.usc.edu/wp-content/uploads/how_to_apply/ADNI_Acknowledgement_List.pdf.

## Abbreviations

SC: Structural connectome
A$$\beta$$: Amyloid-beta
AD: Alzheimer’s disease
HC: Healthy controls
MCI: Mild cognitive impairment
BEI: Balanced excitation-inhibition
FC: Functional connectivity
swFCD: Sliding-window functional connectivity dynamics
phFCD: Phase functional connectivity dynamics
KS: Kolmogorov-Smirnov distance
ADNI: Alzheimer’s Disease Neuroimaging Initiative

## Footnotes

1 https://surfer.nmr.mgh.harvard. edu/fswiki/FreeSurferMethodsCitation

2 https://www.humanconnectome.org/software/fconnectome-workbench

3 http://www.mrtrix.org

4 https://mrtrix.readthedocs.io/en/latest/dwi_preprocessing/dwipreproc.html

5 https://adni.loni.usc.edu/wp-content/themes/freshnews-dev-v2/documents/mri/ADNI3_MRI_Tech_Manual_v2.pdf

6 https://adni.loni.usc.edu/wp-content/uploads/2017/07/ADNI3-MRI-protocols.pdf

7 https://scikit-optimize.github.io/stable/modules/generated/skopt.gp_minimize.html
