# Supplementary material for: Whole-brain modeling of the differential influences of amyloid-beta and tau in Alzheimer’s disease
Source: Alzheimers Res Ther. 2023 Dec 5;15:210. doi: 10.1186/s13195-023-01349-9 (PMC10696890; doi:10.1186/s13195-023-01349-9)
Supplement: Supplementary file 1 — Additional file 1. [file 13195_2023_1349_MOESM1_ESM.zip › Supplemental AT(N) classific.pdf]

Whole-brain modeling of the differential influences of Amyloid-Beta and Tau in Alzheimer's Disease

Gustavo Patow[1,4*], Leon Stefanovski[2,3], Petra Ritter[2,3], Gustavo Deco[4], Xenia Kobeleva[5,6] and for the Alzheimer's Disease Neuroimaging Initiative

SUPPLEMENTARY MATERIAL

AT(N) Classification

We can do the same analysis we performed for Figure 2 in the main manuscript, this time using the AT(N) classification instead of the classification according to the MMSE classification (HC, MCI and AD). As a first step, we computed a simple visualization, in Figure S1-A,B, with the values on the y axis are the SUVRs for each burden (Aβ and tau) and the horizontal axis is the MMSE classification. Just below this plot, at Figure S1-C,D, we can observe the excellent agreement between these values and the average Aβ and tau burdens rendered on a 3D surface.

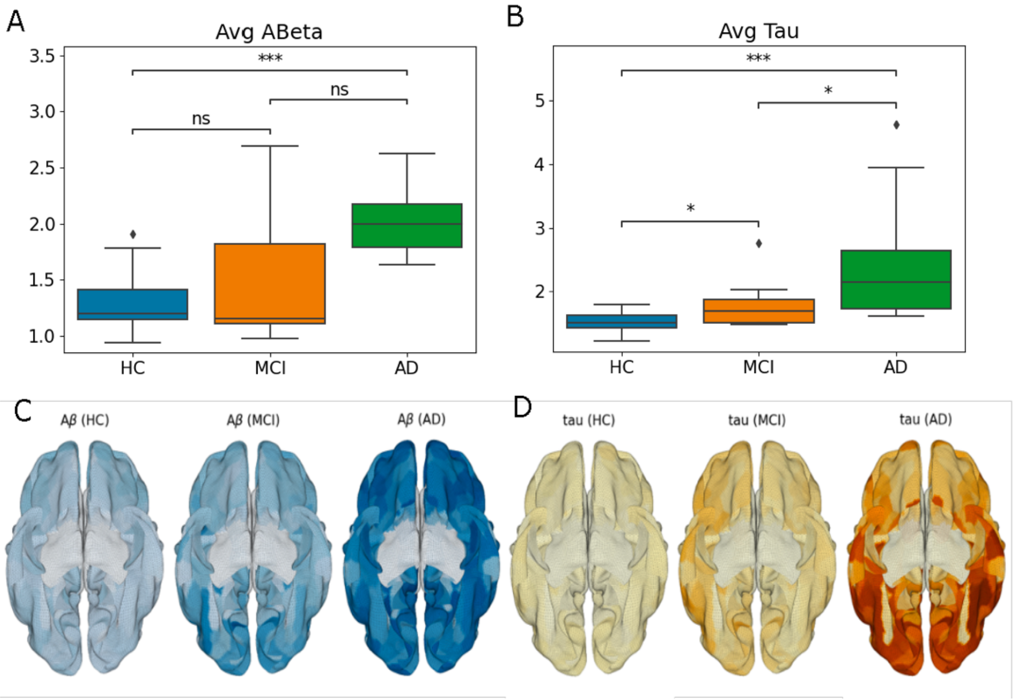

Figure S1: SUVr values for Aβ and tau for each cohort (HC, MCI and AD).

For further classification, as thresholds, we used 0.9 of the average SUVr value for each burden, which results in a threshold of 1.4219 for Aβ, and 1.67 for tau. We want to emphasize that the value for Aβ perfectly matches the value of 1.42 recommended by Jack and co-authors [Jack et al 2017]. However, in general there is some discrepancy in the literature about defining such thresholds for both Aβ and tau, so there is no standard definition for this value [Clark et al 2011, Jack et al 2014, Schöll et al 2016, Jack et al 2017], and even semiquantitative measures are used [Boluda et al 2014, Burke et al 2021], such as CERAD scores [Mirra et al 1991] and Braak staging [Braak and Braak 1991]. As a result, we redistributed our original cohorts into four sets, each with a different number of subjects: A-T-: 15, A-T+: 4, A+T-: 5, and A+T+: 13. As the number for A-T+ and A+T- cohorts is fairly small, it is to be expected to get inconclusive results when comparing with these two cohorts.

We can analyze each case as we did before, for the MMSE classification in the main paper. For A-T-, we observe the same effect as in the original HC case, where the effects of Aβ and tau are close to the homogeneous BEI model, with Aβ presenting a higher prevalence than tau. Again, we can see that Aβ already plays an important role, even in A-T- subjects.

For the A-T+ case, as expected given the low number of subjects in this set, we see a set non-conclusive results, with the combined burden still showing the best values.

Next, the A+T- case presents, despite the limited number of subjects considered, a clear statistical distinction between the computations with the combined burden with respect to all the other cases, but the other combinations show inconclusive results among themselves. Again, in this case Aβ shows the closest agreement with this base value.

Finally, the A+T+ case, we see again that the combined Aβ and tau burdens minimize the computed error, but this time it is tau that shows results the most like the combined one, showing that it is tau the main responsible of the observed behavior at this stage.

As a final remark, we can observe that the A-T- case shows similar behavior as MCIs in MMSE-based controls (with Aβ being more important), and that the A+T+ case shows the same results as MMSE-based AD (with Tau being a bit more important, similar to the original results in Fig2 D,E,F).

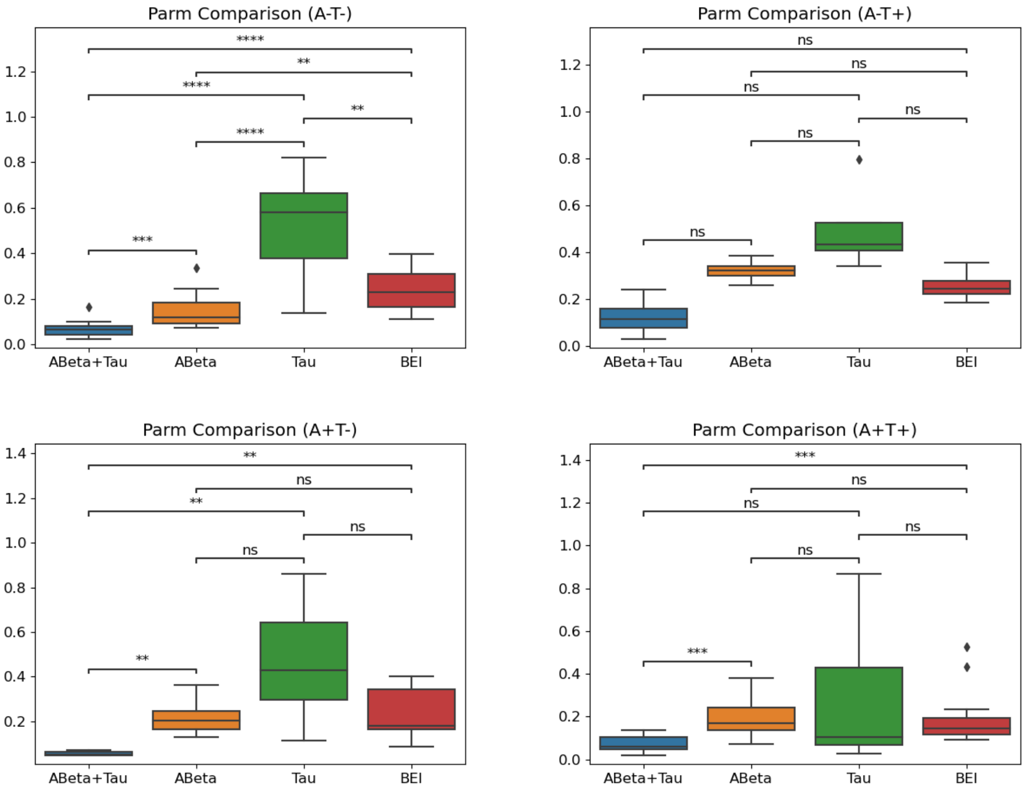

Figure S2: Comparison of the impact of the different burdens in the AT(N) classification (smaller values are better). The plot presents the phFCD (KS distance) for the parameters when optimized together and in isolation, with the homogeneous state as a reference. Again, as in the MMSE case, in all cases, the combined burden outperforms any other model.

Bibliography:

[Braak and Braak 1991] Braak, H., Braak, E. Neuropathological stageing of Alzheimer-related changes. Acta Neuropathol 82, 239–259 (1991). https://doi.org/10.1007/BF00308809

[Mirra et al 1991] S. S. Mirra, A. Heyman, D. McKeel, S. M. Sumi, B. J. Crain, L. M. Brownlee, F. S. Vogel, J. P. Hughes, G. van Belle, L. Berg, participating CERAD neuropathologists, (CERAD) Part II. Standardization of the neuropathologic assessment of Alzheimer's disease, Neurology Apr 1991, 41 (4) 479; DOI: 10.1212/WNL.41.4.479

[Clark et al 2011] Clark, C. M., Schneider, J. A., Bedell, B. J., Beach, T. G., Bilker, W. B., Mintun, M. A., et al. (2011). Use of florbetapir-pet for imaging β-amyloid pathology. JAMA. 305, 275–283. doi: 10.1001/jama.2010.2008)

[Jack et al 2014] Jack, C. R., Wiste, H. J., Weigand, S. D., Rocca, W. A., Knopman, D. S., Mielke, M. M., et al. (2014). Age-specific population frequencies of amyloidosis and neurodegeneration among cognitively normal people age 50-89 years: a cross-sectional study. Lancet Neurol. 13, 997–1005. doi: 10.1016/S1474-4422(14)70194-2)

[Boluda et al 2014] Boluda, S., Toledo, J.B., Irwin, D.J. et al. A comparison of Aβ amyloid pathology staging systems and correlation with clinical diagnosis. Acta Neuropathol 128, 543–550 (2014). https://doi.org/10.1007/s00401-014-1308-9

[Schöll et al 2016] Schöll, M., et al., PET Imaging of Tau Deposition in the Aging Human Brain. Neuron, 2016. 89(5): p. 971-982.). DOI: 10.1016/j.neuron.2016.01.028

[Jack et al 2017] Jack, C.R., Jr., Wiste, H.J., Weigand, S.D., Therneau, T.M., Lowe, V.J., Knopman, D.S., Gunter, J.L., Senjem, M.L., Jones, D.T., Kantarci, K., Machulda, M.M., Mielke, M.M., Roberts, R.O., Vemuri, P., Reyes, D.A. and Petersen, R.C. (2017), Defining imaging biomarker cut points for brain aging and Alzheimer's disease. Alzheimer's & Dementia, 13: 205-216. https://doi.org/10.1016/j.jalz.2016.08.005

[Burke et al 2021] Burke, BT, Latimer, C, Keene, CD, et al. Theoretical impact of the AT(N) framework on dementia using a community autopsy sample. Alzheimer's Dement. 2021; 17: 1879–1891. https://doi.org/10.1002/alz.12348
